# Supplementary material for: A qualitative examination of causal factors and parent/caregiver experiences of non-fatal drowning-related hospitalisations of children aged 0–16 years
Source: PLoS One. 2022 Nov 23;17(11):e0276374. doi: 10.1371/journal.pone.0276374 (PMC9683605; doi:10.1371/journal.pone.0276374)
Supplement: S1 File — (DOCX) [file pone.0276374.s001.docx]

S1 File: Questionnaire to be completed by researcher (based on patient records)

Title of study: NSW Study of drowning and near drowning in children (0-16)

**Demographic information**

1. **Full name of child:**
2. **Date of birth:_______________________________________**
3. **MRN:**
4. **Country of birth:**
5. **Language spoken at home:**
6. **Aboriginal or Torres Strait Islander (circle):**

 No  Yes, Aboriginal

 Yes, Torres Strait Islander  Yes, both Aboriginal and Torres Strait Islander

**Incident details**

1. **Please give a brief description of what happened**

1. **Injury Type:**  Immersion  Submersion
2. **Mechanism of injury:**

 T68: Hypothermia

 T75.1 - Drowning and nonfatal submersion

 W65-W74 Accidental drowning and submersion *(please circle below)*

- W65 Drowning and submersion while in bath-tub
- W66 Drowning and submersion following fall into bath-tub
- W67 Drowning and submersion while in swimming-pool
- W68 Drowning and submersion following fall into swimming-pool
- W69 Drowning and submersion while in natural water, ***Incl.:*** lake / open sea / river / stream
- W70 Drowning and submersion following fall into natural water
- W73 Other specified drowning and submersion
- W74 Unspecified drowning and submersion

 X31 Exposure to excessive natural cold

 X34-X39 Drowning and submersion due to cataclysm

 X71 Intentional self-harm by drowning and submersion

 X92 Assault by drowning and submersion

 V01-V99 Drowning and submersion due to transport accidents

 Y21 Drowning and submersion, undetermined intent

 Other:

 Unknown

1. **How was the patient transported to the hospital?**

 By ambulance from another hospital  By ambulance from where the incident occurred

 Careflight  By the family from where the incident occurred

 Other:

1. **Was Trauma call put out by ED (circle)?** Yes / No
2. **State of child on arrival:**
3. **Was the child intubated (circle)?** Yes / No
4. **By whom (circle)?** Ambulance Officer / At hospital
5. **Was the child admitted to ICU (circle)?** Yes / No
6. **Date of discharge:**
7. **Length of stay:**
8. **ISS Score:**
9. **Outcome:**  Died  Near drowning
10. **Neurological deficits (circle)?** Yes / No / Unknown
11. **Any other comments?**
