# Supplementary material for: A qualitative examination of causal factors and parent/caregiver experiences of non-fatal drowning-related hospitalisations of children aged 0–16 years
Source: PLoS One. 2022 Nov 23;17(11):e0276374. doi: 10.1371/journal.pone.0276374 (PMC9683605; doi:10.1371/journal.pone.0276374)
Supplement: S2 File — (DOCX) [file pone.0276374.s002.docx]

**S2 File: Interview schedule with parent/carer**

Title of study: NSW Study of drowning and near drowning in children (0-16)

**Section 1: Demographic information** *Pre-fill prior to commencing interview using patient records questionnaire*

1. **Name of parent/carer being interviewed: __________**
2. **Full name of child:**
3. **Gender:** Male / Female
4. **Date of Birth:**
5. **Age at incident:**
6. **Address:**
7. **Suburb:**
8. **Phone number:**
9. **Country of birth:**
10. **Language spoken at home:**
11. **Aboriginal or Torres Strait Islander:**

 No  Yes, Aboriginal  Yes, Torres Strait Islander  Yes, both Aboriginal and Torres Strait Islander

**Section 2: Incident details**

1. **Date of incident:____/____/_________ 13. Time of Incident (approx.):____________________________**

**14. Season:** Summer/ Autumn / Winter /Spring

**15. Are you able to tell me about what happened to (*Child’s Name*)?**

**16. Where did the incident occur? (Name of suburb and aquatic location e.g. name of beach, river or public pool):**

**______________________________________________­**

**Incident site (tick)**

 Swimming pool, including inflatable or portable pool *(section 3 also needs to be completed)*

 Public pool  Beach  River/lake/creek

 Bath  Dam  Pond

 Other: ______

**17. Do you remember where the adult carers were at the time of the incident?**

______

**18. How was the child being supervised?**

**** Within arm’s reach in the water **** From a distance while the child was in the water

 Unsupervised, but aware child is in aquatic location

 Unsupervised and unaware the child is in or near water and is unsupervised

**19. Do you recall how long (*Child’s Name*) was under water? ___________________________**

**20. Child found by: 21. Child resuscitated by: ______**

**22. Was an ambulance called (circle):** Yes / No

**23. Have you had any training in CPR? (circle)** Yes/No

If yes, please name most recent year/s: If yes, please name organizations or service of training:

**Section 3: Swimming Lessons:**

**24. Had (Child’s name) participated in swimming / water familiarisation lessons prior to the incident ( (circle):** Yes / No

- If yes, how long had (child’s name) been taking swimming or water familiarisation lessons:
- Were the swimming lessons formal or family instruction:

***Children above 5 years of age:***

**25. Parent’s report of child’s proficiency in swimming (circle):** Good / Average / Poor

**Note: If incident occurred in a pool (including inflatable and portable pools), complete SECTION 4**

**Section 4: Private swimming pool incidents, including inflatable/portable pools**

**26. Who did the swimming pool belong to?**

 Our family pool  Friend’s pool  Grandparent’s pool

 Neighbour’s pool  Other:  **__**

**27. Do you remember what type of swimming pool it was?**

 Permanent above-ground

 Permanent in-ground

 Permanent semi in-ground

 Large inflatable or portable pool (capable of being filled with more than 300mm of water)

 Small inflatable or portable pool (capable of being filled with less than 300mm of water)

**28. Do you know the approximateheight /depth of the pool (cm/m)? __**

**29. Was the pool full at the time of the incident?_______________________________________________________**

If no, how much water would you say was in the pool?**____________________________________________**

**30. In what type of property was the swimming pool located?**

 Department of Housing  Rental property  Owned

**31. How long were you or the occupants living in the house prior to the drowning event?** _____________________________________________________________________________________________

****Note: For inflatable or portable pools, Complete SECTION 5****

**32. Do you recall when the swimming pool was installed?**

 Month and Year: ___________

 Currently approved or being built

 Unknown **(go to question 33)**

**33. If year is unknown, can an estimate be made as per the categories below?**

 Before August 1990 (exemptions exist on older pools installed <August 1990)

 August 1990 - 2000

 2001- June 2010

 After June 2010 (exemptions removed for waterfront, small and large properties)

 Installed or set up within the past 6 months

 I don’t know

**34. Was the swimming pool already installed/set up prior to moving into the property?**

 Yes (when did the family move in):

 No

 Unknown

**Section 5: Inflatable or Portable Pools Only**

**35. How was the inflatable or portable pool purchased?**

 Online  In person from a store  Received as a gift

 Unsure  Other:

**36. Where was it purchased from?**

 Major department store, e.g. Kmart/ Target/ Big W/ Bunning’s etc. (please name): ____________________________

 Other store e.g. one dollar shop (please name):

 EBay

 Other online store (please name):

 Unsure

 Other:

**Section 6: Pool Fencing and Council Approval (swimming pools, including inflatable/portable)**

**37. Did the swimming pool have a fence around it?**

 Yes (**Go to Question 38)**

 No (**Go to Quesiton 39)**

 I don’t know

**38. If yes, what type of fence was it?**

 4 sided isolation fencing

 Fencing including a boundary fence/s as a side

 Fencing including the wall/s of the house as a side

 Other:

**39. If no, did the pool have any exemptions for fencing?**

 Yes – Built prior to August 1990

 Yes – Waterfront property (built prior to 1 July 2010)

 Yes – Small property, less than 230 square metres, (built prior to 1 July 2010)

 Yes – Large property, 2 hectares or over, (built prior to 1 July 2010)

 Yes – Other:

 No exemptions

**40. Do you know if development approval had been obtained from the local council for the pool?** Yes / No

**41. Do you know if the pool has ever been inspected by the local council or a private certifying authority?**

 Yes (Go to question 42)  No (Go to question 43)  I don’t know (Go to question 43)

**42**. **If yes, when was it inspected?**

 When the pool fence was installed  Within the past six months

 Within the past year  Within the past two years

 Within the past three years  Within the past four years

 Five or more years ago  When the house was sold / leased

 Never had the pool fence inspected  Unsure

**43.**  **Can you recall if there were any faults with the pool fence at the time of the incident?**

□ Yes (Go to question 44) □ No □ No fencing

□ I don’t know (Go to question 45)

**44.**  **If yes, please identify below:**

 No temporary fencing around new swimming pool as it’s being built

 Pool fence height less than 1.2m high

 Objects within the Non Climbable Zone (90cm arc from the top of the fence to the ground)

 Vertical gaps in the pool fence too big (>10cm gap between bars)

 Horizontal gaps in the pool fence too small (Bars <90cm apart from each other)

 Gap at the bottom of the fence too big (>10cm from finished ground level)

 Gate not self-closing (without force, from any open position on the gate)

 Gate not self-latching (without force, from any open position on the gate)

 Pool gate swings inwards, towards the pool area

 Pool gate propped open

 Gate latching device location (if not located at least 150cm above ground level, must be located on the inside of the pool fence, at least 15cm below the top of the gate, with a shield fitted around the latch of at least 45cm radius)

 CPR sign not present, ripped or broken

 No lockable lid or fence around the spa pool

 Windows as part of the barrier not compliant (locking device or a security screen has to be present that prevents them from opening more than 10cm)

 Doors as part of the barrier not compliant: (Please specify below)

 Doesn’t self close

 Doesn’t self latch

 Latching device isn’t located at least 150cm off the ground

 Footholds are present wider than 1cm on the door or its frame between the floor and 100cm above the floor

 Pet door present

 Other:

**45. Do you have any advice or recommendations you would give to another parent to prevent a similar incident from occurring?**
