# Supplementary material for: A qualitative examination of causal factors and parent/caregiver experiences of non-fatal drowning-related hospitalisations of children aged 0–16 years
Source: PLoS One. 2022 Nov 23;17(11):e0276374. doi: 10.1371/journal.pone.0276374 (PMC9683605; doi:10.1371/journal.pone.0276374)
Supplement: Attachment — Submitted filename: Response to reviewers.docx [file pone.0276374.s003.docx]

26-09-2022

Senthil Kumaran, MBBS, MD, DNB
Academic Editor
PLOS ONE

Dear Senthil Kumaran,

We thank the reviewer for their work in providing feedback to our manuscript PONE-D-22-17354 entitled “A qualitative examination of causal factors and parent/caregiver experiences of non-fatal drowning-related hospitalisations of children aged 0-16 years” which we submitted for consideration for publication in PLOSONE.

We are pleased to see there is support for our study after revisions. We have indicated below our responses to the reviewer’s comments and also made changes in the manuscript using the track changes functionality in Microsoft Word.

We feel our manuscript is now stronger as a result of these revisions and we hope our manuscript is now ready for publication. It would be an honour to see our work published in PLOSONE.

Yours sincerely,

Boshra Awan

Kids Health Promotion Unit,

Sydney Children’s Hospitals Network

[boshra.awan@health.nsw.gov.au](mailto:boshra.awan@health.nsw.gov.au)

| Suggested revision | Author response |
| --- | --- |
| Journal requirements | |
| 1. Please ensure that your manuscript meets PLOS ONE's style requirements, including those for file naming. The PLOS ONE style templates can be found at  <https://journals.plos.org/plosone/s/file?id=wjVg/PLOSOne_formatting_sample_main_body.pdf> and  <https://journals.plos.org/plosone/s/file?id=ba62/PLOSOne_formatting_sample_title_authors_affiliations.pdf> | Thanks for this feedback. We have ensured as best we can that the submission of the revision meets the requirements of PLOS ONE as per the templates. |
| 2. Please provide additional details regarding participant consent for your retrospective study. In the ethics statement in the Methods and online submission information, please ensure that you have specified what type you obtained (for instance, written or verbal, and if verbal, how it was documented and witnessed). If your study included minors, state whether you obtained consent from parents or guardians. If the need for consent was waived by the ethics committee, please include this information. | Thanks for this feedback. We have added further detail around the participant consent in the methods in the revised manuscript. Please see the revised manuscript to see these changes in context, but in brief, the added text is as follows:  “Parents/caregivers of eligible children were provided with an information sheet and a consent form when they attended hospital. For those parents who were willing to be contacted (i.e., completed the consent form in hospital), parents/caregivers were contacted via phone by the Research Officer at CHW and again invited to participate in the study. This invitation reiterated the nature of the project and the intent for findings to be used to inform drowning prevention and water safety initiatives. Research officers were empathetic and genuine in their approach, to build rapport and trust.  Consent was also verbally obtained from the parent/caregiver of the child via telephone. When the researcher made the telephone call to the parent/caregiver, the researcher asked the question “would you like to be part of this research by answering some questions?”. If they agreed then the researcher commenced asking the questions. If they declined, the researcher thanked them for their time and they ended the call. The parent/caregivers’ consent was recorded on the researcher’s database as “yes” or “no”.” |
| 3. In your Data Availability statement, you have not specified where the minimal data set underlying the results described in your manuscript can be found. PLOS defines a study's minimal data set as the underlying data used to reach the conclusions drawn in the manuscript and any additional data required to replicate the reported study findings in their entirety. All PLOS journals require that the minimal data set be made fully available. For more information about our data policy, please see <http://journals.plos.org/plosone/s/data-availability>.  Upon re-submitting your revised manuscript, please upload your study’s minimal underlying data set as either Supporting Information files or to a stable, public repository and include the relevant URLs, DOIs, or accession numbers within your revised cover letter. For a list of acceptable repositories, please see <http://journals.plos.org/plosone/s/data-availability#loc-recommended-repositories>. Any potentially identifying patient information must be fully anonymized.  Important: If there are ethical or legal restrictions to sharing your data publicly, please explain these restrictions in detail. Please see our guidelines for more information on what we consider unacceptable restrictions to publicly sharing data: <http://journals.plos.org/plosone/s/data-availability#loc-unacceptable-data-access-restrictions>. Note that it is not acceptable for the authors to be the sole named individuals responsible for ensuring data access.  We will update your Data Availability statement to reflect the information you provide in your cover letter. | Thank you for this query. We are unable to upload the minimal dataset underlying this analysis due to strict ethical requirements. We have expanded on this and changed the point of contact for seeking access to the data s per PLOS’ agreements in our revision. Our revised text in the Data Availability Statement now reads:  “Underlying data used in this study cannot be uploaded due to ethical constraints surrounding the sensitive nature of the data. Those interested in learning more about the data and potentially accessing the data, can contact [the](mailto:the) Hunter New England Human Research Ethics Committee via email (hnehrec@hnehealth.nsw.gov.au) and quote reference number 13/05/15/4.06.” |
| 4. Please review your reference list to ensure that it is complete and correct. If you have cited papers that have been retracted, please include the rationale for doing so in the manuscript text, or remove these references and replace them with relevant current references. Any changes to the reference list should be mentioned in the rebuttal letter that accompanies your revised manuscript. If you need to cite a retracted article, indicate the article’s retracted status in the References list and also include a citation and full reference for the retraction notice. | Thank you. We have checked our reference list and made minor amendments to formatting, including adding websites where online only material is available. We have not referenced any retracted studies. Please see the revised manuscript. |
| Reviewer 1 | |
| Reviewer #1: The manuscript PONE-D-22-17354 describes a qualitative study exploring parent/caregiver experiences of non-fatal drowning related hospitalisations of their children. The major strength of the paper is that it examines the experiences of parents for an important supervision behaviour in efforts to inform future preventative strategies. The main issues I have with the paper lie in (1) an absence of detail of the ethical procedures followed for a sensitive topic among a vulnerable group and (2) the lack of rationale for exclusion of children with a pre-existing health condition/disability. These minor points, along with other issues, are detailed below. | Thanks very much for your work in reviewing our manuscript. We have actioned each of your points below and in the manuscript. No further action required. |
| Abstract and Introduction  1. It is unclear what ICD is referring to in the Abstract. | We have now explained this acronym in full, which is a well-known health classification system (International Classification of Diseases). These edits have necessitated minor edits elsewhere to bring the abstract back within the allowable word limit. Please see the revised manuscript. |
| 2. The wording in the Abstract of “are to be made” is awkward and should be replaced with wording such as “should be made”? | Thank you for this suggestion. We agree and have revised as suggested. Please see revised manuscript. |
| 3. In the Introduction, a stronger rationale for the role of a qualitative approach in this context should be offered in addition to simply its absence historically (p. 3). | Agree. We have revised the last paragraph of the introduction to include the following sentence as to the value of qualitative research such as that presented in our manuscript beyond it being a research gap. The sentence we have added reads as follows: “Qualitative data can provide rich detail on non-fatal drowning incidents including valuable insights from parents and caregivers to inform future child drowning prevention efforts.” |
| Method and Results  4. Why were children with a disability or pre-existing health conditions in particular excluded from the study? (p. 5). Please provide a rationale. | Thank you for this query. You raise an important point. We do acknowledge that drowning risk is elevated among children with a disability and/or pre-existing health condition. In excluding children with a disability or pre-existing medical condition from this study, our rationale was that these children form a unique sub-population who likely have different risk factors for drowning from children without disability or pre-existing medical conditions. In that respect, children with these conditions deserve a separate unique study with specific questions asked and analysis conducted to explore the impact of such conditions on drowning risk and the circumstances leading up to a non-fatal drowning.  We have revised our exclusion criteria in the methods and it now reads as follows:  “Children who were admitted due to incidents which, on closer examination, were incorrectly coded as an immersion/submersion incident and were not in-fact drowning-related, were excluded from the study. Children with pre-existing health conditions or a disability were also excluded from this study. The rationale behind decision was that greater attention should be paid to this sub-group and different tools used to capture insights that increase drowning risk among this unique population. It is however, acknowledged as a limitation of this research and we recommend dedicates studies with parents/caregivers of children with a disability or pre-existing medical condition in the future. “ |
| 5. “pre-exiting” should re “pre-existing” on p. 5. | Thank you for drawing our attention to this typographical error. It has been amended in the revision. |
| 6. Can more information please be provided about the consent process for parents being contacted? Did the parents agree to be contacted for research purposes as part of the care they received at the hospitals or was a waiver of consent received in this instance to cover the screening process of hospital records? Given the sensitive nature of the topic among a potentially vulnerable cohort, more detail about this process would be useful for the reader (p. 5). | Thank you for this query. We have further revised the detail in the manuscript to better explain the consent process. The following is the revised text:  “Parents/caregivers of eligible children were provided with an information sheet and a consent form when they attended hospital. For those parents who were willing to be contacted (i.e., completed the consent form in hospital), parents/caregivers were contacted via phone by the Research Officer at CHW and again invited to participate in the study. This invitation reiterated the nature of the project and the intent for findings to be used to inform drowning prevention and water safety initiatives. Research officers were empathetic and genuine in their approach, to build rapport and trust.  Consent was also verbally obtained from the parent/caregiver of the child via telephone. When the researcher made the telephone call to the parent/caregiver, the researcher asked the question “would you like to be part of this research by answering some questions?”. If they agreed then the researcher commenced asking the questions. If they declined, the researcher thanked them for their time and they ended the call. The parent/caregivers’ consent was recorded on the researcher’s database as “yes” or “no”. “ |
| 7. What is meant by “to.. ultimately ensure successful recruitment” as it sounds a little coercive with the current wording? Perhaps re-word? (p, 5). | Thank you for this suggestion. The process was the opposite of coercive as researchers were incredibly mindful of the sensitive nature of this research, but we agree this wording is clumsy. We have deleted this phrase “and ultimately ensure successful recruitment.” Please see the revised manuscript. |
| 8. The qualitative analysis undertaken was fairly superficial but likely appropriate given the research question of interest and the fact the study did not comprise an indepth exploration of the effects of the likely traumatic experience on parents/caregivers. | Thank you for these comments. No further changes required. |
| Discussion  9. Could the Discussion include slightly more consideration of the result relating to brief immersions and the ramifications for training/education strategies? | Thank you for this suggestion. We have added the following to further identify the importance of education on supervision to prevent even a brief immersion which can still pose danger to children: “In addition, many caregivers mentioned the issue of a brief immersion. Despite the perceived short period of time the child was immersed in water, such incidents resulted in children being admitted to hospital, no doubt causing significant stress on child and caregivers. Such findings are an important reminder of the need to prevent the immersion incident in the first place, including through effective parental or caregiver supervision (13). Drowning prevention organisations and advocates should consider the inclusion of parental and caregiver information about the dangers of even a brief immersion, and the importance of supervision as a primary preventive strategy.” |
| 10. Would it be useful to draw more comparisons of the findings of the present study with those that have examined fatal drownings re identified similarities/differences? | Thanks for this suggestion. We have linked to coronial data on fatal child drowning in the sections of the discussion on CPR, swimming, barriers and supervision. Most findings are similar to coronial data for this age group, with the exception of parent/caregiver reported information on swimming ability or experience in learn to swim lessons, which is rarely reported in coronial data for this age group. Please see the revised manuscript. |
| Recommendation:  The stated points above are easily addressed and the manuscript is a worthwhile contribution to the extant literature. The paper contributes usefully to our understanding of this important parental supervision/child safety topic. | Thank you very much for your work in reviewing our manuscript and suggesting these minor revisions. No further action required. |
